# Touchless Heart Rate Monitoring from an Unmanned Aerial Vehicle Using Videoplethysmography

**DOI:** 10.3390/s23167297

**Published:** 2023-08-21

**Authors:** Anna Pająk, Jaromir Przybyło, Piotr Augustyniak

**Affiliations:** Department of Biocybernetics and Biomedical Engineering, AGH University of Krakow, 30 Mickiewicz Ave., 30-059 Krakow, Poland; annapaja@agh.edu.pl (A.P.); przybylo@agh.edu.pl (J.P.)

**Keywords:** noninvasive measurements, remote measurements, contactless, videoplethysmography, RGB video, unmanned aerial vehicle, heart rate

## Abstract

**Motivation**: The advancement of preventive medicine and, subsequently, telemedicine drives the need for noninvasive and remote measurements in patients’ natural environments. Heart rate (HR) measurements are particularly promising and extensively researched due to their quick assessment and comprehensive representation of patients’ conditions. However, in scenarios such as endurance training or emergencies, where HR measurement was not anticipated and direct access to victims is limited, no method enables obtaining HR results that are suitable even for triage. **Methods**: This paper presents the possibility of remotely measuring of human HR from a series of in-flight videos using videoplethysmography (VPG) along with skin detection, human pose estimation and image stabilization methods. An unmanned aerial vehicle (UAV) equipped with a camera captured ten segments of video footage featuring volunteers engaged in free walking and running activities in natural sunlight. The human pose was determined using the OpenPose algorithm, and subsequently, skin areas on the face and forearms were identified and tracked in consecutive frames. Ultimately, HR was estimated using several VPG methods: the green channel (G), green-red difference (GR), excess green (ExG), independent component analysis (ICA), and a plane orthogonal to the skin (POS). **Results**: When compared to simultaneous readings from a reference ECG-based wearable recorder, the root-mean-squared error ranged from 17.7 (G) to 27.7 (POS), with errors of less than 3.5 bpm achieved for the G and GR methods. **Conclusions**: These results demonstrate the acceptable accuracy of touchless human pulse measurement with the accompanying UAV-mounted camera. The method bridges the gap between HR-transmitting wearables and emergency HR recorders, and it has the potential to be advantageous in training or rescue scenarios in mountain, water, disaster, or battlefield settings.

## 1. Introduction

Nowadays, there are numerous methods for measuring human vital parameters. Broadly speaking, these methods can be classified as invasive and noninvasive. Invasive methods are primarily used in hospitals and exclusively by trained staff. This type of monitoring requires a specific controlled environment and incurs significant costs mainly due to the single-use equipment such as needles, wires, bandages, etc. Noninvasive measurements involve capturing the desired signal without breaking the skin tissue, making them safer for the patient’s health. However, the drawback of such measurements is the ease of signal distortion, which can result in uncertain or incorrect readings.

The variety of noninvasive methods is substantial. Human vital signs can be collected using wearable devices [1], clothing (e.g., intelligent garments), or standalone touchless devices and systems embedded in household appliances [2].

One dynamically developing method for touchless pulse measurement is videoplethysmography (VPG). It is based on the optical properties of blood tissues. The blood flow in the vessels causes skin color changes that are invisible to the human eye but detectable by electronic sensors. Light absorption in the blood depends, among other factors, on its oxygenation. The shift of the absorption spectrum maximum between oxygen-rich blood and blood with low oxygen content is distinct. This difference allows the heart rate to be determined from a series of images recorded with a standard video camera. In [3], a video sequence was analyzed using the RGB color palette, and in [4], the video was presented in grayscale. Proper lighting selection is important during VPG measurements to enhance the reliability of pulse results [4].

To obtain more accurate results, various preprocessing and analysis methods have been investigated. A review of known and currently used methods is presented in [5]. Most analyses are based on the RGB color palette (extracting the green component, calculating the green-red difference, or excess green). Another approach, unaffected by the color palette, employs independent component analysis [3].

The motivation behind the proposed method for measuring physiological parameters is to enable health monitoring for individuals for whom measuring pulse through contact methods is not possible. Without challenging the performance of wearable on-skin devices, we demonstrate that measurements using non-contact sensors can expand the range of rapid triage to scenarios where wearables are impractical or infeasible. Non-contact monitoring becomes a unique option when providing medical supervision for individuals without accessible physical contact. For instance, utilizing in-flight VPG allows for remote assessment of accident victims’ conditions in scenarios such as disasters, terrorism, combat fields, mountain and potentially water rescues, where direct access is difficult, unsafe, or time-consuming, and the need for measurement was unforeseen. Victims of a mass accident will not all have wearable wristbands, and even if they do, connecting them poses additional challenges to address. In such cases, aerial VPG measurements with UAVs open the path for efficient, accurate, and speedy remote monitoring and triage. This principle also applies to the synchronous monitoring of multiple individuals in scenarios like running marathons, endurance trainings, or other mass events.

Our method aims to simultaneously track multiple individuals outdoors using UAVs. Field recording necessitates consideration of various factors (lighting, silhouette scale, movement type, unstable camera), introducing diverse challenges and research questions to address. The feasibility studies presented in this paper address the following research inquiries:

- Is it feasible to reliably monitor the heart rate of a moving individual using a video sequence captured in-flight with a camera attached to a UAV?
- How can we accurately track and identify different subjects in natural motion?
- How should we handle scale variations in the human silhouette due to varying subject-to-sensor distances? When a person is away from the camera, his or her face occupies fewer pixels in the image, which reduces the signal-to-noise ratio.

By combining a silhouette detection algorithm based on deep neural networks (OpenPose [6]) with a method for tracking multiple objects simultaneously [7], we have developed the capability to identify facial and other body parts and monitor them over a sufficient duration to measure the pulse. Utilizing 4k resolution video largely mitigates the silhouette scale issue, as physiological information is represented across more skin pixels.

The main contributions of this article are as follows:

- We present a method for measuring the pulse in individuals without a wearable pulse recorder and in the absence of direct contact.
- We introduce a method for detecting and tracking people in videos recorded by a UAV-mounted camera.
- We propose a method of skin area identification using a human pose estimation algorithm.
- We conduct an evaluation of selected VPG-based heart rate estimation algorithms on videos from an unstable aerial camera.

## 2. Related Work

Studies conducted by [8,9] emphasize the significance of obtaining human vital parameters remotely. Ali Al-Naji et al. in [8] utilized a UAV to record one-minute videos of each participant at a resolution of 1920 × 1080 and a frame rate of 60 frames per second. For analysis, they considered the last 30 seconds of each recording. Heart and breath rates were determined using the iPPG signal from the face. In the first scenario, the participant’s face remained motionless; in the second scenario, the participant had to make various facial expressions and rotate their head; finally, in the third scenario, the measured person spoke. None of these scenarios appear to justify the use of a UAV. The algorithms studied in this paper were Complete Ensemble Empirical Mode Decomposition with Adaptive Noise (CEEMDAN) and Canonical Correlation Analysis (CCA). Despite the application of advanced methods, the obtained results were similar to those from well-known Independent Component Analysis and Principal Component Analysis.

The footage recorded using a UAV was the subject of research in [9]. The authors analyzed the heart rate rPPG signal from the participants’ faces. The videos were recorded for 30 seconds, and the volunteers remained seated and motionless. CHROM [10] and POS [11] methods were employed for HR detection. The average RMSE was high (14.3 bpm), rendering the results unsuitable for health surveillance practice. This article also addresses the challenge of active image stabilization during video recording. In contrast to experiments involving immobilized subjects, the authors assert that stabilizing the UAV-mounted camera is crucial, especially in mountain rescue operations due to frequent changes in wind and weather conditions. The authors posit that integrating a three-axis gimbal with the UAV could enhance the accuracy of acquisition and signal-to-noise ratio.

In addition to HR measurements, there have been attempts to read respiratory rates based on UAV recordings. In [12], the video was synchronized with cardiorespiratory activities using reference devices such as pulse oximeters and piezoelectric breathing belts. Due to the high influence of noise in the VPG signal, the authors utilized methods like full decomposition in empirical modes with adaptive noise (CEEMDAN) and canonical correlation analysis (CCA), among others. A study involving 15 healthy volunteers demonstrated that the results obtained from the proposed method were superior to those from Independent Component Analysis (ICA) and Principal Component Analysis (PCA).

Machine learning was also employed in VPG measurements. In [13], the authors proposed an end-to-end video transformer architecture called PhysFormer for remote physiological measurement. This architecture primarily comprises a video temporal difference transformer backbone. The transformer architecture [14], initially proposed for modeling sequential data in the field of natural language processing (NLP), was adapted to incorporate the vision transformer (ViT) [15], which was recently introduced for image classification. According to the authors, this architecture can effectively model the long-range spatio-temporal relationships required for reliable rPPG measurement. Experiments were conducted for three types of physiological signals: heart rate (HR), heart rate variability (HRV), and respiration frequency (RF). Four benchmark datasets were employed: VIPL-HR [16], MAHNOB-HCI [17], MMSE-HR [18], and OBF [19]. These databases predominantly consist of face videos captured in various conditions.

The authors of [20] proposed a convolutional neural network denoted as HR-CNN for estimating the heart rate of a subject in a video sequence. The network’s input was a sequence of cropped images containing the subject’s face (192 × 128 resolution), and the output was the predicted heart rate value. The network comprises two components. The first component (extractor) was trained on the PURE database [21], while the second component (HR estimator) was trained on different databases separately. The evaluation databases (MAHANOB [17], COHFACE [22], and ECG-Fitness dataset collected by the authors of the article) primarily consist of videos recorded in indoor environments.

In [23], the authors present a novel convolutional attention network (DeepPhys) that simultaneously learns a spatial mask to detect the appropriate regions of interest and recover the heart rate and respiration signals. The architecture of the proposed network consists of two parts based on a VGG-style convolutional neural network (CNN), which shares the spatial masks. The current video frame and the normalized difference between frames are provided as inputs to the appearance and motion models of this network. The input images were cropped to include subjects’ faces (with resolutions ranging from 130 × 130 to 492 × 492 pixels). The authors tested the proposed method on four datasets, including two RGB datasets collected during experiments, one infrared dataset, and the MAHANOB-HCI [17] dataset. The dataset used by the authors comprises videos recorded in a controlled studio setup and an indoor environment.

Article [24] presents an on-device optical cardiopulmonary vital sign measurement approach using multi-task temporal shift convolutional attention network (MTTS-CAN). It leverages the previous work of the authors [23] but extends it to include temporal dependencies beyond consecutive frames using different architecture of the convolutional network (Hybrid-CAN). Moreover, the proposed solution can work in real time. Authors evaluate their system on two large public datasets: AFRL [25] and MMSE-HR [18]. Datasets used in evaluation consists of videos recorded in controlled indoor lighting environment.

All methods based on deep networks ([13,20,23,24]) have been trained on videos containing a single person in a controlled indoor environment (though with various lighting and movement conditions). These methods either assume an image containing only the face in the right proportions as input for deep networks or require the use of a separate face detector. Additionally, most of these approaches require a method of training deep networks (transfer learning) to adapt them to a different video database. Incorporating them into our framework would necessitate significant modifications to these algorithms. Therefore, they are not included in this manuscript.

VPG measurements are based on the analysis of changes in skin color due to heartbeats and breathing. An important aspect of these measurements is the difference in skin tones caused by variations in the ethnic origin of the subjects. In [26], the differences in results obtained from the study of Caucasians (with fair skin), Mongolians, and individuals of African descent (with darker complexions) were analyzed. The analysis employed a PCA-based algorithm for five color spaces: red channel, green channel, red chrominance channel, RGB color space, and RG color space. Based on the research, it was determined that the amount of melanin in the skin alters light absorption by the skin, thereby affecting the measurement results. For individuals with fair complexions, an analysis based on the green channel is recommended, while for those with darker skin tones, the optimal color is red. Acknowledging this, our study analyzed results obtained from volunteers with consistent skin tones, and thus no differentiation was applied in the analysis.

## 3. Methods

### 3.1. Video Recording

The video was recorded outdoors with a camera attached to a three-axis gimbal on a UAV (DJI Mavic Air 2, maximum take-off weight 570 g). The camera resolution was 4K Ultra HD (i.e., 3840 × 2160 pixels). A licensed UAV operator maneuvered the aircraft to maintain a height of approximately 2.5 m at a distance of 5 to 7 m from the volunteer, ensuring that the camera captured the person being measured. The volunteer faced the front of the aircraft, where the camera was mounted. As the volunteer attempted to approach, the aircraft moved backward to maintain the set distance. Initially, the volunteer walked and then gradually transitioned to running, attempting to keep up with the UAV. A Polar H10 single-lead ECG-based chestband served as a wearable reference heart rate sensor. The time from the video and the chestband was synchronized to validate the accuracy of heart rate estimation from the video.

There are several papers related to the validity of the Polar H10 device. For example, the Polar white paper [27]. Additionally, according to [28], the HRV measurements derived from the Polar H10 chest strap device demonstrate strong agreement and a small bias when compared with ECG recordings, making it a recommended choice for practitioners.

All participants were healthy (none had chronic diseases that would pose risks during the measurement). Before the measurement, they familiarized themselves with the procedure and completed the necessary documentation. Informed consent was obtained from all human subjects. Participants were instructed to move at their own comfortable rhythm and speed, and they were not exposed to any stressful conditions. The device used for collecting the ground truth pulse rate was battery-powered and commercially available for personal use.

During the measurement, the size of the human pose changed due to inevitable variations in distance between the human and the UAV. A smaller human pose size resulted in a lower signal-to-noise ratio. Regions where the skin was visible consisted of a small number of pixels. To mitigate this issue and maintain a safe distance between the aircraft and the human, the footage in this study was recorded in 4K resolution.

### 3.2. Overview of the Algorithm

**Figure 1:**
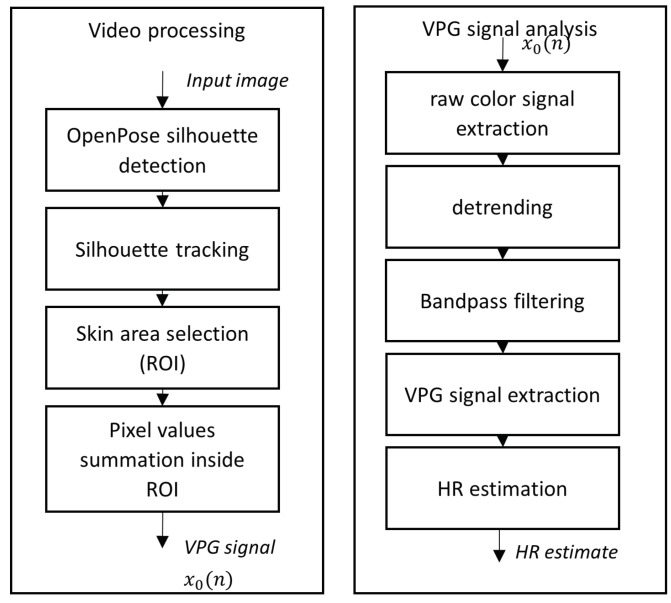
An overview of the architecture of the proposed method.

An illustration of the architecture of the proposed method is presented in Figure 1.

**Figure 2:**
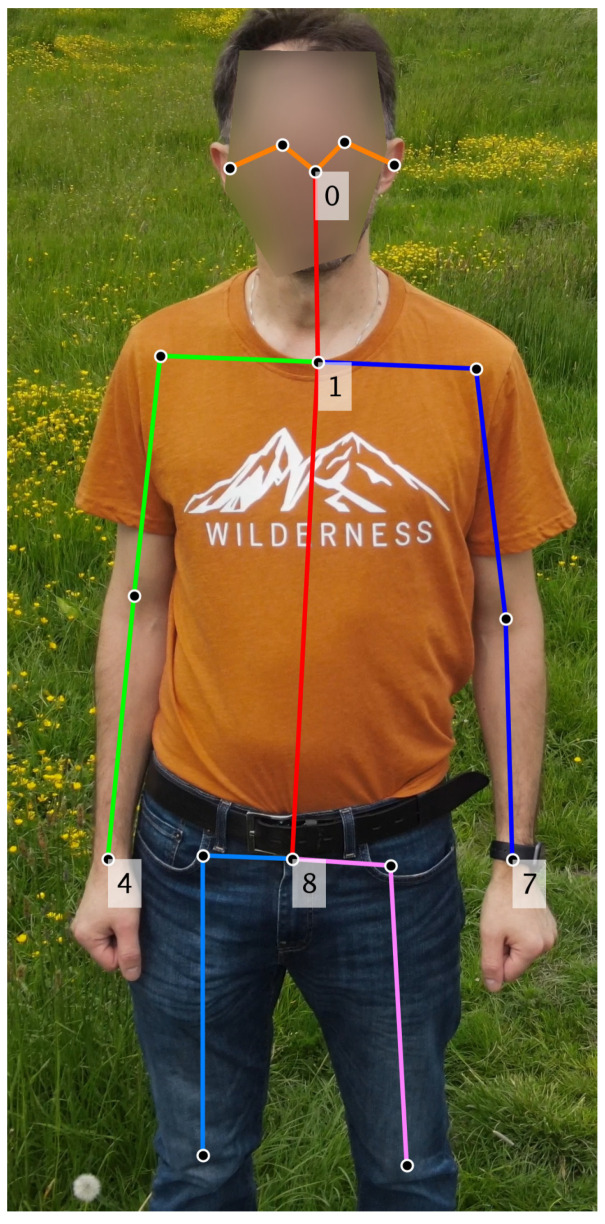
The silhouette points of the model BODY_25 used to determine whether it was accurately detected (points 0, 1, 4, 7, 8) and used to determine the center of the silhouette (mean of points 1 and 8) in the tracking algorithm.

The data processing was carried out in two stages. In the first method, the input video underwent analysis using image processing techniques: (1) area of interest tracking and (2) skin area detection to extract the videoplethysmographic signal. To ascertain the face or forearm positions, the OpenPose [6] algorithm was initially employed to identify points on the human silhouette. Subsequently, based on the positions of these selected points (depicted in Figure 2), the area of interest (ROI) was defined. Finally, within the selected ROI, the sum of pixels for each RGB component of the image was computed. In scenarios involving monitoring two participants simultaneously, proper indexing of the detected individuals was essential to prevent mismatching their heart rates. For this purpose, the multi-object tracking algorithm [7] was utilized. The average location of selected points on the silhouette was assumed as the center of the tracked object.

Using the results of tracking the silhouette in the source video sequences, the sequences were segmented into fragments containing only one accurately detected silhouette from those visible in the image. Further analysis was solely conducted on fragments that met a minimum length criterion (i.e., no shorter than the FFT transform used in the subsequent step). For each video frame *n* within these video fragments, the sum of pixels calculated in the areas of interest constituted a raw color videoplethysmographic signal sample denoted as $x_{0}\left(n\right)={\left[R\left(n\right);G\left(n\right);B\left(n\right)\right]}^{T}$. This signal was subsequently processed in the second stage of analysis.

The second stage of analysis aimed to estimate the pulse from the $x_{0}\left(n\right)$ signal and is detailed in Section 3.4.

### 3.3. Area of Interest Tracking

During the motion in the camera’s field of view, the position of the volunteer was constantly changing, due to the movement of both the human and the UAV. In the recorded video sequence, it was necessary to track the human pose. For this purpose, the OpenPose algorithm [6] was applied.

Utilizing the experimentally determined parameters, the pose was correctly detected and tracked using OpenPose BODY_25 model. The posture was detected based on points representing the head, abdomen and hands (see Figure 2). The primary challenge involved tracking a single posture without any significant points transitioning beyond the region of interest, such as onto another human or into the background. To address this issue, an algorithm was implemented to identify exceptional distance values between points of the posture in two consecutive video frames or the disappearance of a crucial posture point. In such situations, the detected pose was considered lost, and the process was restarted by initiating a new search for the human pose.

Another challenge in analyzing human poses was the lack of indexing of detected positions. When two people were recorded simultaneously, a simple algorithm may randomly mismatch their labels. To fix this problem, a multiple object tracking technique and the Kalman filter were used [7]. Both methods are conceived for tracking and indexing objects based on their last known location. The prediction is most precise for constant velocity or acceleration of the measured object. The speed of volunteers can be approximated to be constant or in some cases—slowly growing. The multiple object tracking algorithm was run with following options:

- *prediction method*: Kalman constant velocity filter,
- *detection to track cost functions and elimination of unlikely matches*: detection to track cost function, representing the cost of an unassigned track or detection (distance between track and detection position) was set to 20 pixels,
- *assign detections to track*: Munkres variant of the Hungarian bipartite matching algorithm was used,
- *track management*: tracks that have been invisible for too many consecutive frames (>5) were deleted, and recently created tracks that have been invisible for too many frames (>2) were deleted.

### 3.4. Skin Area Detection

The detection of skin area posed a challenge due to the rapid camera shakes. Initially, the algorithm had to track the human posture and subsequently, based on the contour, segment skin regions—specifically, the face and arms—that were suitable for VPG analysis. The OpenPose algorithm met these requirements and was employed for this purpose.

**Figure 3:**
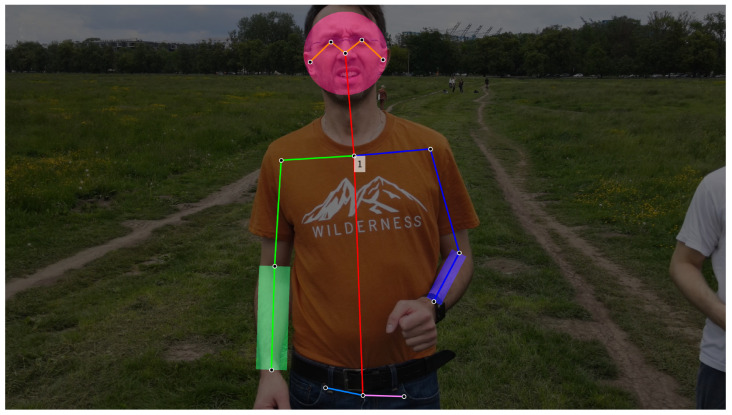
An Example of detected volunteer’s face and forearms combined with segmentation markers calculated by OpenPose algorithm in a video frame.

After extracting the human posture from the video, the exposed skin areas were identified. The areas where most skin was anticipated included the face and both forearms. An approximate circle was employed to represent the face contour, while rectangles were used for approximating the forearm regions. The size of these regions of interest (ROI) was determined as a percentage of the pose dimensions. An example of the detected skin regions is illustrated in Figure 3.

### 3.5. Heart Rate Estimation

After segmenting the skin regions, the skin color in these areas was analyzed in each consecutive image from the sequence according to the VPG paradigm using the following steps (refer to Figure 1):

- Extracting the raw color signal $x_{0}\left(n\right)$ by summing pixel values inside regions of interest (ROI)—the face or forearms.
- Detrending using an algorithm based on mean centering with scaling (MCwS) [8].
- Signal filtering with a bandpass filter in the range of plausible pulse values (40–180 bpm).
- Interpreting color variation in consecutive frames as a VPG signal using five selected alternative methods: green channel (G), green-red difference (GR), excess green (ExG) [5], independent component analysis (ICA) [3], and a plane orthogonal to the skin (POS) [11].
- Normalizing and filtering the pulse signal.
- Calculating the heart rate using the periodogram power spectral density estimate.

The following algorithm parameters were used:

- *MCwS detrending window length*: 0.4 s (i.e., 12 frames for 30 frames per s (fps) video),
- *bandpass filter*: order = 32, lower cutoff frequency = 0.75 Hz (45 bpm), upper cutoff frequency = 3.0 Hz (180 bpm),
- *heart rate estimation using periodogram power spectral density estimate*: FFT length = 512 datapoints (i.e., 17.07 s of video at 30 fps).

**Figure 4:**
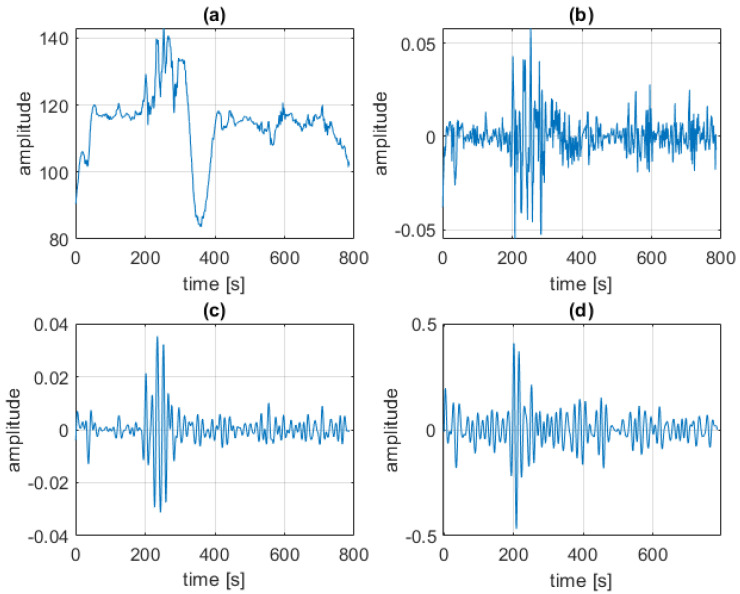
An example of results from each step of video processing pipeline: (**a**) raw signal (G channel); (**b**) detrended signal; (**c**) filtered signal; (**d**) VPG signal (POS).

Sample results from each step of video processing are shown in Figure 4.

## 4. Results

**Figure 5:**
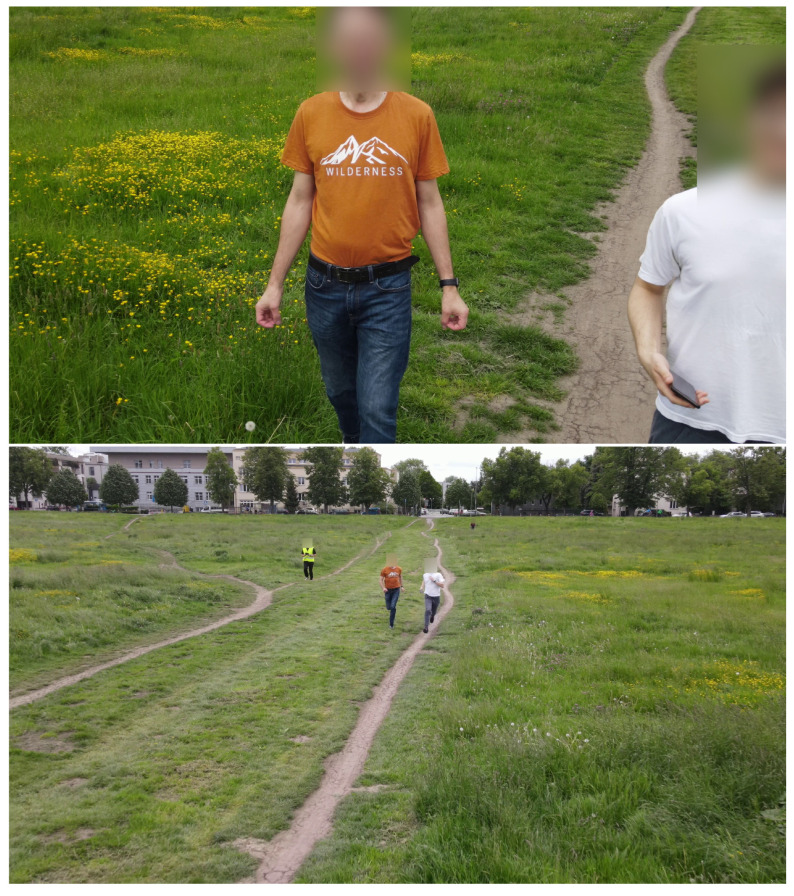
Example scenes from recordings. Two volunteers in the foreground are simultaneously free running; the person behind in the yellow vest is the UAV operator.

Ten video clips of varying lengths, ranging from 3 min and 25 s to 4 min and 50 s, were recorded. The recordings were captured on both sunny and cloudy days at a city meadow park. Consequently, alongside the study participants, random silhouettes of other individuals are also present, justifying the implementation of a multi-person tracking algorithm. The total number of participants in the study was seven, although some individuals were recorded multiple times. Participants were instructed to either take a short walk or run along a designated path. The UAV operator made efforts to keep the moving participants within the camera’s field of view, although this was not always feasible. As a result, the size of the silhouettes in the recordings exhibited significant variation (refer to Figure 5).

Since participants did not consistently appear in the recordings, it became necessary to utilize not only a silhouette detection algorithm (OpenPose) but also to track multiple individuals simultaneously. Following this stage, the source videos were divided into shorter sequences so that the examined person appeared in all frames. As a result, a regularly sampled discrete videoplethysmographic signal could be obtained. All video fragments were manually reviewed and verified by a human expert in terms of the correctness of silhouette detection. Depending on the FFT length used, a minimum of 512 frames were required. Therefore, for further analysis, only the fragments of sufficient length for determining the pulse were retained. For the selected FFT length, the blood pulse frequency resolution equals $\frac{Fs}{NFFT}=\frac{30}{512}=0.0586$ Hz (equivalent to 3.5156 bpm). The resulting number of usable video fragments was 32.

For each video fragment, a VPG signal was formed using the algorithm described in Section 3.5 (involving pixel summation in the skin areas, detrending, filtering, VPG signal extraction, heart rate estimation). A summary of all detection results for individual fragments and VPG extraction methods is included in Appendix A.

The performance of the proposed method is measured using difference metrics between the VPG-calculated pulse and the ground truth (gt) captured with the reference wearable recorder. The mean value of systematic error and standard deviation (SD) of the differences, along with 95% limits of agreement (±1.96 SD), and root mean squared error (RMSE, in accordance with Equation (1)) were calculated for the estimated heart rates from all video segments using all selected VPG algorithms. When comparing the accuracy of the pulse rate measured from various areas, the region of interest (ROI) selected on the head skin area yields smaller errors than the two forearms. RMSE errors for respective VPG color spaces are compared in Table 1.

(1) $$RMSE=\sqrt{\frac{1}{K}\sum_{k=1}^{K}{\left(hr_{gt}-hr_{0}\right)}^{2}}$$

where *K*—number of video fragments.

**Figure 6:**
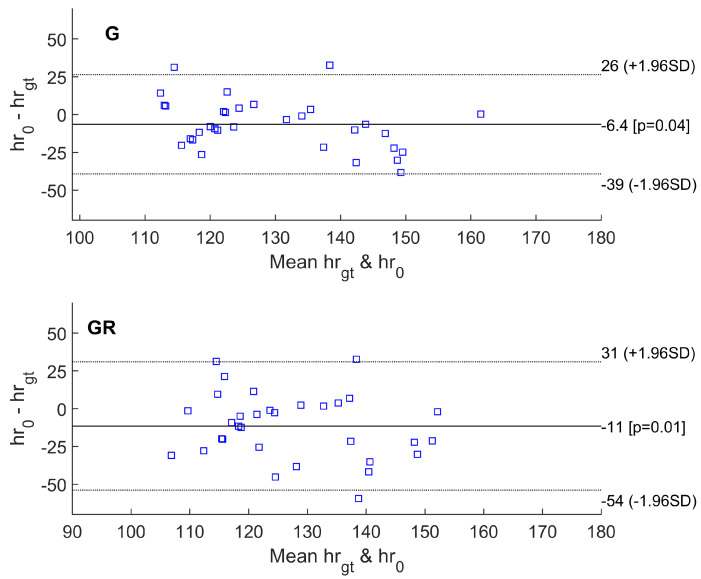
Bland–Altman plots of difference between heart rate measurements obtained by the reference method and heart rates estimated using G and GR methods.

**Figure 7:**
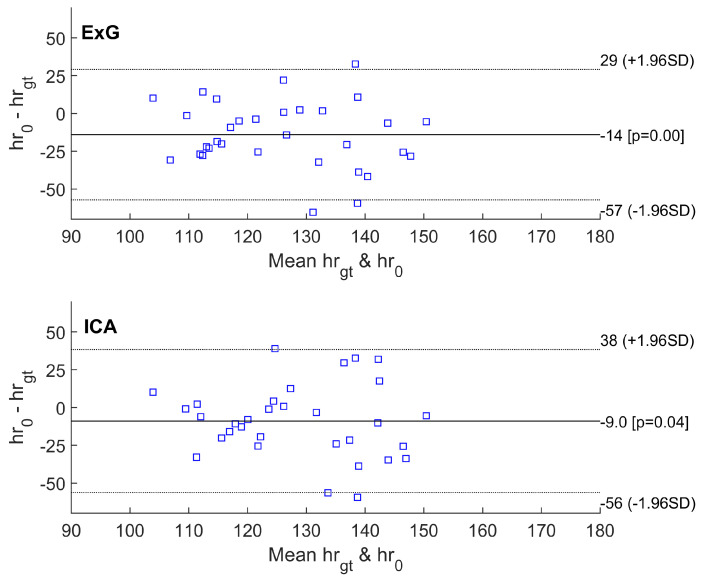
Bland–Altman plots of difference between heart rate measurements obtained by the reference method and heart rates estimated using ExG and ICA methods.

**Figure 8:**
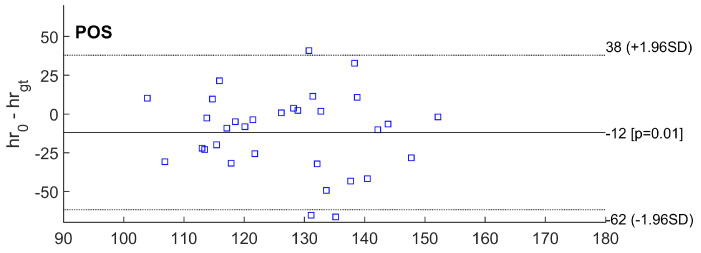
Bland–Altman plots of difference between heart rate measurements obtained by the reference method and heart rates estimated using POS method.

Other statistics are shown on Bland–Altman plots (Figure 6, Figure 7 and Figure 8). The abbreviations used in the figures are as follows:

- $hr_{gt}$—ground truth heart rate from PolarH10 device,
- $hr_{0}$—results of heart rate detection.

The Bland–Altman plots showed the lowest mean bias of −6.4 bpm for the basic G method. Most values fell within the range between a lower limit of −39 bpm and an upper limit of +26 bpm in the 95% confidence interval.

Similarly, the smallest RMSE error was achieved for the simplest algorithm based on the analysis of the green channel (G). For 14 out of 32 video sequences, at least one of the tested methods exhibited an error smaller than the FFT resolution (approximately 3.5 bpm). For the G and GR methods, an error of less than 3.5 bpm occurred for six video fragments. For the ICA and POS algorithms, an error of less than 3.5 bpm occurred for five video fragments. For the ExG method, the fewest sequences had an error of less than 3.5 bpm.

## 5. Discussion

The advantage of using a camera mounted on a UAV to monitor human pulse is primarily the flexibility of such a solution. Depending on the needs, it is possible to monitor various scenarios, such as athletes during exercise, individuals injured in challenging conditions (in mountainous areas), or elderly people moving in public spaces. Importantly, this measurement method is contactless and does not require the use of pre-prepared wearables or diagnostic devices. The utilization of human pose estimation algorithms enables the identification of skin areas crucial for pulse measurement. In the same equipment setup, it is also possible to monitor a person’s movement patterns, providing insights into conditions like musculoskeletal diseases.

The OpenPose algorithm allows for real-time multi-person 2D pose estimation from images. Ablation studies were conducted by the authors of the original algorithm [6], comparing their approach with several other methods. In cases where one silhouette obscures another or when silhouettes are close to each other, we observed that some shape points may be covered or assigned to the wrong person. This issue was partially addressed with an algorithm that detects significant differences in the distances between posture points in two adjacent video frames (as described in Section 3.3). To mitigate this problem in future studies, we suggest considering more recent multiple object tracking algorithms that can handle such cases. When participants were not present in the recordings throughout the entire duration, it was necessary to divide the source videos into smaller fragments (with a minimum number of frames set to FFT length) to ensure that the examined person appears in all frames and HR measurement is possible (Section 4). Interpolation of missing samples of the pulse wave is worth considering in future works.

Furthermore, our aim includes extracting VPG information from different body parts, thereby enabling the estimation of other cardiovascular parameters such as pulse transient time.

As for the accuracy of the measurement of physiological parameters, the conducted research reveals the need for further data analysis and identification of sources of interference possibly affecting the result. Large discrepancies in the results of individual pulse estimation methods may originate from the influence of different sources. Different estimation methods are sensitive to disparate types of disruption. Confirmation of this hypothesis, however, requires further research.

Based on the results of previous research published in [4], it can be concluded that one of the sources of errors is high compression of the image during the capturing process (H264, MPEG-4 AVC, part 10 codec, bitrate = 103332 kb/s, yuv420p). At the frame size of 3840 × 1920 pixels and a speed of 30 fps, the bitrate only allows for 0.416 bits per pixel representation (equivalent to a compression ratio of nearly 20 times when compared to a standard 8-bit dynamic resolution). To mitigate this issue, faster data carriers, smaller frames, or lower frame rates were necessary. Unfortunately, the first solution is costly and unique to ensure minimal impact on video quality.

Another potential source of measurement errors may be the incorrect separation of the skin area. In some video sequences, it was observed that determining the skin area as a circular contour, based on the distance of the detected body shape points, led to the inclusion of pixels from neighboring background areas. A possible direction for improvement could be the utilization of semantic segmentation algorithms for identifying skin pixels.

Another research and engineering challenge to address is how to process image data in real-time. Embedded systems that can be placed on a drone typically have limited computing power due to weight and battery power constraints. Therefore, using overly complex calculation methods raises performance concerns. Fortunately, HR estimation methods such as G, ExG, ICA, and POS are not computationally demanding. The OpenPose method, despite being based on deep networks, can operate in real time. According to the authors, the inference time of the OpenPose algorithm is invariant to the number of people in the image. However, the following question arises: can it be executed on an embedded system mounted on a UAV? Another potential solution for processing real-time video data is transmitting the data over a wireless network to a computer with adequate computing power. However, this approach necessitates addressing the effective transmission of a substantial amount of data (4k video) with minimal compression (to avoid loss of signal quality).

Also, in this paper we did not conduct research on the impact of illumination conditions on measurement accuracy. The measurement conditions outside were variable (sunny days, cloudy days). The study of the influence of lighting on the measurement was carried out in our previous work [4].

## 6. Conclusions

Based on the results of the present study and the studies [8,9], the feasibility and reliability of measuring human vital parameters using UAV-mounted sensors are confirmed. This research area is particularly important for scenarios involving difficult access to the person being measured (e.g., traffic accidents and mountain rescues). The remote assessment of a victim’s condition justifies prompt assistance and expedites the triage procedure. With the use of the human pose tracking proposed in this manuscript, there is a possibility to measure more than one victim simultaneously.

While conducting measurements and data analysis, the following challenges had arisen: (1) high image compression (which causes errors in the analyzed signal), (2) obstacles in real-time data transmission (due to large file sizes), (3) limited access to computational power and energy supply in the case of analysis performed by an embedded system operating in-flight. The possibility to eliminate difficulties with wireless data sending requires further research (for example, evaluating the use of the 5G network). Further research should be focused on more efficient human pose detection, real-time measurements, and assessment of dependencies on weather conditions (such as the presence of clouds, wind, etc.).

An important direction for future research will also involve adapting and evaluating other pulse detection algorithms, including HR-CNN [20], DeepPhys [23], TS-CAN [24], and PhysFormer [13].

In future research, the use of deep network skin detection is also planned. This network can detect and track measurement areas checked in the first frame. During preliminary tests, the method proposed in [29] shows better results than ours in skin regions delineation and tracking due to the use of accurate free shape-based approximation of body areas instead of our simple geometrical figures: the face modeled with a circle and forearms with rectangles.

## Figures and Tables

**Table 1:** Comparison of VPG extraction algorithms - the RMSE error.

		RMSE [bpm]		
**G**	**GR**	**ExG**	**ICA**	**POS**
**17.7**	24.2	25.8	25.4	27.7

## Data Availability

Not applicable.

## Appendix A. Summary of Results for All Video Fragments and VPG Extraction Algorithms

Summary of all detection results for individual fragments and VPG extraction methods. Labels used in the table columns are:

- video no.—number of continuous video strip
- person id—identifier of the volunteer
- numframes—number of consecutive video frames in the strip
- $hr_{gt}$—ground truth heart rate from PolarH10 device,
- $\Delta hr=\left|hr_{gt}-hr_{0}\right|$—absolute error,

**Table A1:** Summary of all detection results for individual fragments and VPG extraction methods.

Video No.	Person Id	Numframes	${\mathrm{hr}}_{\mathrm{gt}}$ [bpm]	Δhr [bpm]				
				**G**	**GR**	**ExG**	**ICA**	**POS**
video 1, part 1	male0	785	124.2	11.7	**1.1**	18.7	**1.1**	8.1
video 1, part 2	male1	575	122.3	4.3	30.9	30.9	4.3	30.9
video 1, part 3	male0	674	121.7	**1.4**	9.2	9.2	29.5	9.2
video 1, part 4	male1	768	125.4	9.4	19.9	26.9	12.9	19.9
video 1, part 5	male0	1406	124.0	8.0	11.5	22.1	8.0	22.1
video 1, part 6	male1	622	121.1	**2.0**	5.0	5.0	12.5	5.0
video 1, part 7	male0	652	124.9	15.9	12.4	23.0	15.9	23.0
video 1, part 9	male1	898	126.3	10.3	27.9	27.9	31.9	3.8
video 2, part 1	male1	655	115.1	15.0	11.5	22.0	6.1	**2.6**
video 2, part 2	male0	524	125.8	20.3	**2.7**	**0.8**	**0.8**	**0.8**
video 2, part 6	male0	522	123.3	6.8	3.8	3.8	10.8	3.8
video 3, part 1	female1	538	105.2	14.3	21.3	14.3	38.9	21.3
video 3, part 2	female1	1641	134.5	**1.0**	25.6	25.6	25.6	25.6
video 3, part 3	female1	616	127.7	8.2	**2.4**	**2.4**	32.8	**2.4**
video 4, part 1	female1	1576	133.4	**3.3**	3.7	10.8	**3.3**	10.8
video 4, part 2	female1	1266	159.3	22.2	22.2	25.7	25.7	43.3
video 5, part 1	female2	1306	131.9	26.4	**1.7**	**1.7**	19.4	**1.7**
video 5, part 2	female2	1250	161.9	24.8	21.3	28.3	56.4	28.3
video 6, part 1	male2	2928	110.3	5.7	**1.4**	**1.4**	**2.2**	40.8
video 6, part 2	male2	1751	133.7	**3.4**	6.9	14.2	17.5	31.8
video 6, part 3	male2	1054	163.8	30.2	30.2	65.4	33.7	65.4
video 7, part 1	male2	1215	148.2	21.6	21.6	32.2	21.6	32.2
video 7, part 2	male2	2700	147.2	10.1	38.3	20.7	10.1	10.1
video 8, part 1	male3	2943	122.0	32.7	32.7	32.7	32.7	32.7
video 8, part 2	male3	2144	147.1	6.5	45.2	6.5	24.1	6.5
video 8, part 3	male3	1083	161.3	**0.4**	41.8	41.8	34.8	41.8
video 9, part 1	male4	1384	98.9	31.2	31.2	10.1	10.1	10.1
video 9, part 2	male4	5330	125.7	16.7	20.2	20.2	20.2	11.5
video 10, part 2	female2	1991	153.1	12.5	**2.0**	5.5	5.5	**2.0**
video 10, part 3	female2	1753	158.3	31.7	35.2	38.7	38.7	49.3
video 10, part 4	female2	1756	168.4	38.4	59.5	59.5	59.5	66.5
video 10, part 5	female2	866	109.9	6.1	9.6	9.6	**0.9**	9.6
